# Genome-Wide Identification of the AGC Kinase Family in Tetraploid Potato (*Solanum tuberosum* L.) Cultivar ‘Qingshu No. 9’ and Functional Analysis of *StD6PK* in Response to Late Blight (*Phytophthora infestans*)

**DOI:** 10.3390/plants14243818

**Published:** 2025-12-15

**Authors:** Yifan Zhou, Chunna Lv, Yihan Zhao, Yuting Bao, Fang Wang

**Affiliations:** 1 Academy of Agriculture and Forestry Sciences, Qinghai University, Xining 810016, China; 2 Qinghai Provincial Key Laboratory of Potato Breeding, Qinghai University, Xining 810016, China

**Keywords:** potato ‘Qingshu No. 9’, gene identification, *StD6PK* gene, late blight, gene expression analysis

## Abstract

The AGC kinase family is crucial for regulating plant disease resistance, integrating hormone signals, managing reactive oxygen species (ROS) metabolism, and maintaining redox balance. However, research on AGC kinases in Solanaceae plants is limited, and the functions of most AGC kinases remain unidentified. Using the tetraploid potato (*Solanum tuberosum* L.) cultivar ‘Tingsha No. 9’, we conducted a genome-wide identification of the AGC gene family and profiled transcript responses to late-blight (*Phytophthora infestans*) stress. Additionally, we examined the subcellular localization and characterized the phenotypic responses of overexpression lines of the late-blight–responsive kinase *StD6PK* under late-blight stress. A total of 141 AGC family members were identified in ‘Qingshu No. 9’, categorized into eight subfamilies. This classification includes one cultivar-specific subfamily that was previously unrecognized, as well as 50 AGC family members within subfamily 1. AGC family members had significant differences in physicochemical characteristics and most of which were located in the nucleus. AGC family members are distributed on 46 chromosomes, with the largest number of chromosome 11 and the least number of chromosome 7. Gene duplication is dominated by whole-genome duplication (WGD) and segmental duplication. Ka/Ks values of all collinear pairs are less than 1. Purification selection drives family evolution in a long evolutionary process. Its promoter is rich in light-responsive, hormone-responsive, and stress-responsive elements, and its expression varies significantly in tissues; and some genes are highly expressed in specific organs. RNA-seq analysis revealed that 78.1% of the members responded to late-blight stress, and the expression levels of the selected eight subfamily members all showed significant increases or decreases after inoculation with late blight. *StD6PK* (*Soltu.Q9.Chr04_A40011450.g*) was strongly induced at 48~72 h, and its expression level at 72 h was 5.7 times higher than that at 0 h. Stable transformation of potato demonstrated that overexpression of *StD6PK* could enhance the resistance of potato to late blight, with subcellular localization revealing its nuclear localization characteristic. This study was the first time to complete the identification of AGC family genome of tetraploid potato ‘Qingshu No. 9’, reveal its evolution and expression characteristics, clarify the response characteristics of *StD6PK* to late blight, and provide insights into the evolutionary and functional basis of the AGC kinase gene family in potato late blight resistance mechanisms, while supplying genetic resources to accelerate the development of late blight-resistant germplasm.

## 1. Introduction

Protein kinase-mediated protein phosphorylation is one of the main ways in which organisms regulate cell function [1]. The AGC (cAMP-dependent protein kinase, cGMP-dependent protein kinase, and protein kinase C) family of protein kinases is a group of serine/threonine protein kinases, including cAMP-dependent protein kinase A (PKA), cGMP-dependent protein kinase G (PKG), and phospholipid-dependent protein kinase C (PKC) [2]. The AGC kinase family is highly conserved in eukaryotes, and its members regulate cell cycle, metabolic pathways, and stress response by phosphorylating downstream target proteins [3]. AGC kinases are particularly diverse in plants and play important roles in disease resistance regulation, hormone signal integration, ROS metabolism, and redox balance [4,5].

The AGC kinase family has been studied to varying degrees in different plants, most extensively in *Arabidopsis thaliana*, but research in Solanaceae is still lacking. AGCs were identified in *Arabidopsis thaliana* and divided into 7 subfamilies with 39 members. PDK1 subfamily members play important roles in controlling auxin polar transport, signal transduction, and plant stress responses to pathogens [6,7]. Members of the S6K subfamily have been shown to respond to biological stress and light signals, and activation of S6K is regulated by plant hormones [8,9,10,11]. The IRE subfamily has been shown to regulate root hair growth in *Arabidopsis thaliana*, and IRE in *Medicago truncatula* is thought to be associated with the growth of nodule infection thread [12,13]. Mutation of the *NDR1* gene in *Arabidopsis thaliana* inhibited the resistance response of three resistance proteins, RPS2, RPM1, and RPS5, to the *Pseudomonas syringae* DC3000 strain, and overexpression of *NDR1* in *AtNDR1* mutants increased plant resistance to the pathogen [14,15]. AGC1 is the most abundant AGC gene family and plays an essential role in controlling root hair tip growth, pollen tube polar growth, and auxin polar transport [16,17,18,19,20,21]. AGC2 subfamily members have been shown to regulate phytoalpin secretion and disease resistance [22] via the phosphorylation transporter PDR6. Two members of the AGC2-related subfamily (*PHOT1* and *PHOT2*) mediate multiple physiological responses to UV/blue light, and play important roles in chloroplast movement, stomatal opening, phototropism, dark-induced leaf senescence, and enhanced drought resistance in Arabidopsis thaliana [23,24,25,26,27,28].

While studies in the model plant Arabidopsis thaliana have demonstrated that the AGC kinase gene family plays pivotal roles in regulating cell cycle progression, metabolic pathways, stress responses, and critical disease resistance processes through the phosphorylation of downstream target proteins, systematic investigations into this family within the Solanaceae particularly in potato (*Solanum tuberosum* L.), ranked as the fourth most important global food crop remain largely unexplored [29,30,31]. The most harmful global threat to sustainable potato production is late blight caused by *Phytophthora infestans* [32,33,34,35]. The epidemic of potato late blight caused by *Phytophthora infestans* in the 19th century led to a major famine in Ireland, killing one million Irish people and displacing more than two million people [36]. It is estimated that global losses due to late blight are approximately USD 12 billion [37,38,39] per year.

Previous studies have shown that some members of the AGC kinase family are associated with potato late blight [11]. However, the function of AGC kinase in potato is still unclear. Therefore, this study aims to perform a genome-wide identification of the AGC kinase family in ‘Qingshu No. 9’ potato (*Solanum tuberosum* L.) and analyze its functional role in the response to late blight through stable genetic transformation.

## 2. Materials and Methods

### 2.1. Materials and Processing

In this study, the tetraploid potato ‘Qingshu No. 9’ and *Nicotiana benthamiana* were used as research materials. ‘Qingshu No. 9’ was utilized for RNA extraction and genetic transformation, while *Nicotiana benthamiana* served as the primary experimental system for subcellular localization studies. ‘Qingshu No. 9’ [40] was bred by the Institute of Biotechnology, Academy of Agriculture and Forestry Sciences, Qinghai Province. ‘Qingshu No. 9’ exhibits high resistance to late blight in field conditions, but its resistance decreases when evaluated via in vitro leaf assays for late blight [41]. Its skin was purple-red, its flesh was yellow and partly red, and it was resistant to late blight. ‘Qingshu No. 9’ and *Nicotiana benthamiana* came from the Institute of Biotechnology, Academy of Agriculture and Forestry Sciences, Qinghai Province. *Nicotiana benthamiana* was grown in a light incubator (25 °C; light 16 h/dark 8 h; light intensity 10,000 lx; humidity 80%), potato ‘Qingshu No. 9’ was planted in a glass greenhouse of Qinghai University (altitude 2312 m; latitude 36.728139) and sown with peat soil and vermiculite of 3:1.

### 2.2. Identification of AGC Gene Family in Tetraploid Potato ‘Qingshu No. 9’

A total of 39 Arabidopsis AGC proteins downloaded from the TAIR database (https://www.arabidopsis.org/) were used to construct hidden Markov model (HMM) files, and BLASTp (TBtools, v2.376) was performed on the genome of ‘Qingshu No. 9’ using these 39 proteins [42]. Candidate genes were further validated by scanning for conserved domains using InterPro (https://www.ebi.ac.uk/interpro/, accessed on 25 June 2025). The names of AGC genes identified are shown in Table S1.

### 2.3. Phylogenetic Analysis, Protein Conserved Motif Analysis, and Physico-Chemical Characterization of Potato AGC Protein

MEGA X (v10.2) was used to construct phylogenetic trees and protein multiple sequence alignments [43]. Multiple sequence alignments were performed using ClustalW (MEGA, v10.2), and phylogenetic trees were constructed according to the maximum likelihood method. Bootstrap was set to 1000, and other parameters were set to default values. Evolutionary tree visualization was performed using Evolview (https://www.evolgenius.info/, accessed on 25 June 2025) [44]. The amino acid number, isoelectric point, and molecular weight of the *StAGC* protein were determined by TBtools (v2.376). WoLF PSORT (https://wolfpsort.hgc.jp/, accessed on 25 June 2025) was used to analyze the subcellular localization of AGC genes [45,46]. TBtools was used to visualize the location of the *StAGC* gene on chromosomes. Intron/exon location information of the *StAGC* gene was mapped according to the GFF file, and the MEME Suite (https://meme-suite.org/meme/, accessed on 25 June 2025) performed conservative motif analysis. Motif search was set to 10, and the rest of the parameters were set to default values. Finally, TBtools was used to generate pictures [47].

### 2.4. Collinearity Analysis in AGC

The replication events of the *StAGC* gene were analyzed using McScanX (TBtools, v2.376) software, a built-in feature of TBtools (v2.376) software (Table S3), and the nonsynonymous (Ka) and synonymous (Ks) replacement rates of duplicate gene pairs and Ka/Ks values were calculated (Table S2). In addition, the whole-genome files and GFF3 files of tomato, Arabidopsis thaliana, maize, and rice were downloaded from Ensembl Plants (https://plants.ensembl.org/index.html, accessed on 25 June 2025), and collinearity analysis of AGC genes of different species and potato was performed.

### 2.5. Analysis of Cis-Acting Elements

A sequence 2000 bp upstream of the *StAGC* gene was extracted from the tetraploid potato genome of ‘Qingshu No. 9’ and submitted to the online tool PlantCARE (https://bioinformatics.psb.ugent.be/webtools/plantcare/html/, accessed on 25 June 2025) for predicting potential cis-acting elements in the sequence and visualized using TBtools [48].

### 2.6. Tissue Expression Profile Analysis

RNA-seq data extracted FPKM values for 6 tissues from the paper attachment published by Wang, F. et al. [42], followed by mapping gene expression profiles, including 6 tissues (root, stem, leaf, flower, pedicel, and stolon) of potato variety ‘Qingshu No. 9’, and tissue expression heat maps using TBtools.

### 2.7. Expression Analysis of AGC Kinase Family Under Late-Blight Stress in Potato

To investigate the expression patterns of the AGC kinase gene family at 0 h, 24 h, 48 h, and 72 h following inoculation with late blight, we retrieved raw RNA-seq data from BioProject PRJNA7283373 [49]. Quality control of the raw sequencing reads was conducted using FastQC, and the filtered clean reads were aligned to the reference genome of Solanum tuberosum (‘Qingshu No. 9’, https://ngdc.cncb.ac.cn/gwh/submit/27179/step?status=1, accessed on 25 June 2025) with HISAT2 for transcript assembly. Transcriptome assembly and quantification were executed using StringTie, employing the merge option to create a consolidated transcriptome annotation. Subsequently, the FPKM (fragments per kilobase of transcript per million mapped reads) values for each gene were calculated to assess expression levels. The expression levels of 141 gene family members are detailed in Table S5.

### 2.8. qRT-PCR Analysis of StAGCs Under Late-Blight Stress in Potato

After potato ‘Qingshu No. 9’ grew to 60 cm in the greenhouse, it was inoculated with *Phytophthora infestans* P21202 (physiological race: 1.2.3.4.5.6.7.8.9.10.11). After 0 h, 24 h, 48 h, and 72 h of inoculation, parts of the periphery of the lesions on the diseased leaves were taken and frozen in liquid nitrogen, and the samples were stored in −80 °C refrigerator. Total RNA was extracted from the leaves at each stage using a plant RNA extraction kit (Takara Biotech, Beijing, China) and then reverse-transcribed into cDNA (Takara Biotech, Beijing, China). *StEF1α* [50] was selected as the housekeeping gene for qPCR analysis due to its stable expression levels under various stress conditions, including Phytophthora (late blight) infection. Three biological replicates and three technical replicates were performed for each experiment, and relative gene expression was calculated using the 2^−∆∆Ct^ method [51]. A histogram was drawn using Origin, and the qRT-PCR primers used are shown in Table S2.

### 2.9. Subcellular Localization of StD6PKConstruction and Localization Observation of StD6PK Subcellular Carriers

The CDS of *StD6PK* without a stop codon was inserted into vector 35S GFP-1300 by the double enzyme digestion method. After sequencing confirmation, *StD6PK*-35S GFP-1300, 35S GFP-1300, and nuclear positioning marker (RFP) plasmids were transformed into competent Agrobacterium GV3101-P19. The mixture of target vector and localization vector was injected into tobacco leaves at a ratio of 7:3, and then cultured in the dark for 2 days [52]. Fluorescence was observed by a confocal microscope (Nikon, Tokyo, Japan). Primers used for gene cloning and vector construction are supplemented in Table S4.

### 2.10. Generation of Transgenic StD6PK Potato Plants

Total RNA was isolated from potato ‘Qingshu No. 9’ tissue-cultured seedlings utilizing the Plant Total RNA Extraction Kit (Takara Biotech, Beijing, China). Subsequently, the RNA was reverse-transcribed into cDNA following the guidelines of the FastQuant Kit (Takara Biotech, Beijing, China). The coding sequence (CDS) of *StD6PK* was obtained from the NCBI website. Subsequently, specific primers were developed to amplify the segment encompassing Xma I and BamH I restriction enzyme sites. Post-enzymatic cleavage, *StD6PK* was integrated into the overexpression vector PRI101 employing T4 DNA ligase for vector assembly (Figure S1). The resultant construct, PRI101-*StD6PK*, was introduced into *DH5α* Escherichia coli competent cells (Takara Biotech, Beijing, China). Verification of the presence of the inserted fragment was conducted using specific primers and kanR primers (Figure S2). DNA sequencing was performed on the colonies of Escherichia coli that tested positive, and the colonies with correct sequencing were selected for preservation and propagation.

Potato ‘Qingshu No. 9’ was utilized for the production of transgenic plants. The recombinant vector PRI101-*StD6PK* was introduced into Agrobacterium tumefaciens GV3101-p19 for the transformation of stem segments, following the methodology outlined by Bouaziz [53] (Figure S3). Regenerated plants were cultivated on MS medium supplemented with 100 mg/L Timentin at 22 °C. Positive identification was achieved by amplifying the specific kanamycin resistance fragment (Figure S4). To evaluate the response of *StD6PK* to late blight, stably transformed potato plants were grown in pots for 15 d under conditions of 25 °C with a 16 h/8 h light/dark cycle. Screening was conducted using quantitative real-time PCR (qRT-PCR). *StActin* served as the reference gene due to its stable expression [54,55], which is a standard practice for housekeeping genes. Lines exhibiting elevated transcript levels of *StD6PK* were selected for further assays. To investigate whether *StD6PK* regulates the expression of *StPR*1, *StPR5*, *StSOD*, and *StPOD* genes, this study measured the relative expression levels of these genes in WT and OE lines using qRT-PCR, with *StEF1α* serving as the internal reference gene. All primers utilized in this study are detailed in Table S4.

### 2.11. Inoculation of Phytophthora Infestans Zoospore Fluid, Trypan Blue, and DAB and NBT Staining

To assess the resistance phenotype of *StD6PK*-OE potato plants against late blight in *StD6PK*-OE potato plants, a zoospore suspension of *Phytophthora infestans* isolate P21202 (pathotype: R1–R11) at a concentration of 4 × 10^4^ spores/mL was used for inoculation. Fifteen mature leaves from both the wild-type (WT) and two OE lines were collected for the experiment. Each leaf was inoculated with a 50 μL aliquot of the zoospore suspension, avoiding the midrib, followed by 24 h of dark incubation at 18 °C with the adaxial side up. Leaves were then inverted and maintained under a 16 h light/8 h dark photoperiod at 18 °C. The method for preparing *Phytophthora infestans* sporangial suspensions and post-inoculation management for whole plants was identical to that used for detached leaves. Plants were inoculated with *P. infestans (Phytophthora infestans)* using a hand-held spray bottle, applying 10 mL of sporangial suspension per plant to ensure uniform coverage. Phenotypic assessments, DAB staining, and NBT staining were carried out at 3 d and 5 d post-inoculation (dpi), while trypan blue staining and mycelial observation were performed at 5 dpi. DAB and NBT staining procedures were conducted following the guidelines of the DAB Chromogenic Kit (Solarbio Science and Technology Co., Ltd., Beijing, China; Cat. No. DA1010-10) and the Plant Tissue Reactive Oxygen Species (ROS) Detection Kit (Solarbio Science and Technology Co., Ltd., Beijing, China; Cat. No. G4816), respectively. Trypan blue staining was performed as outlined by Tian [56]. Leaves at 5 dpi were submerged in a 0.5% trypan blue solution for 15–20 min, treated with absolute ethanol for clarification, and subsequently imaged. Mycelial examination was carried out utilizing an optical microscope (OLYMPUS-BX51, Tokyo, Japan).

## 3. Results

### 3.1. Identification of AGC Gene Family Members in Potato ‘Qingshu No. 9’ and Analysis of Their Physico-Chemical Properties

**Figure 1:**
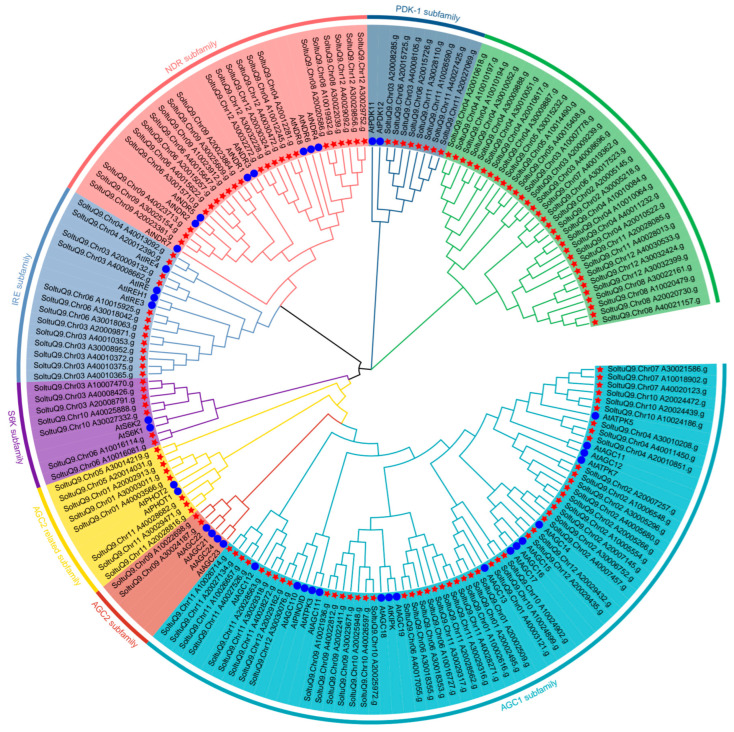
Phylogenetic relationship between potato (*Solanum tuberosum* L.) ‘Qingshu No. 9’ and Arabidopsis AGC protein. The blue circle in the inner circle is the AGC gene of Arabidopsis thaliana, and the red pentagram is the AGC gene identified in potato. Different colors in the outer circle represent different subfamilies, and the order from the open green outer circle is new subfamily, PDK-1 subfamily, NDR subfamily, IRE subfamily, S6K subfamily, AGC 2R subfamily, AGC2 subfamily, and AGC1 subfamily.

In this study, 141 AGC family members were identified in ‘Qingshu No. 9’, and a phylogenetic tree was constructed with AGC gene family protein sequences of *Arabidopsis thaliana* (Figure 1). According to the classification of *Arabidopsis thaliana*, they can be divided into 8 subfamilies, among which the AGC1 subfamily has the most members, with 50 members. AGC gene family members have amino acid residues ranging from 226 to 2002, molecular weights ranging from 26,638.12 to 223,356.3, isoelectric points ranging from 4.82 to 9.41, and most family members are localized in the nucleus (Table S1). In order to understand the developmental relationships among *StAGC* gene family members more clearly, phylogenetic trees were constructed using AGC protein sequences from *Arabidopsis thaliana* and potato (Figure 1). Cluster analysis showed that AGC1 had 50 members, the largest number, and AGC2 had 2 members, the smallest number. In addition, a new subfamily was observed in ‘Qingshu No. 9’, which is independent of the seven subtribes of Arabidopsis thaliana and has 23 members.

### 3.2. Analysis of AGC Gene Family Structure and Conserved Motifs in ‘Qingshu No. 9’

**Figure 2:**
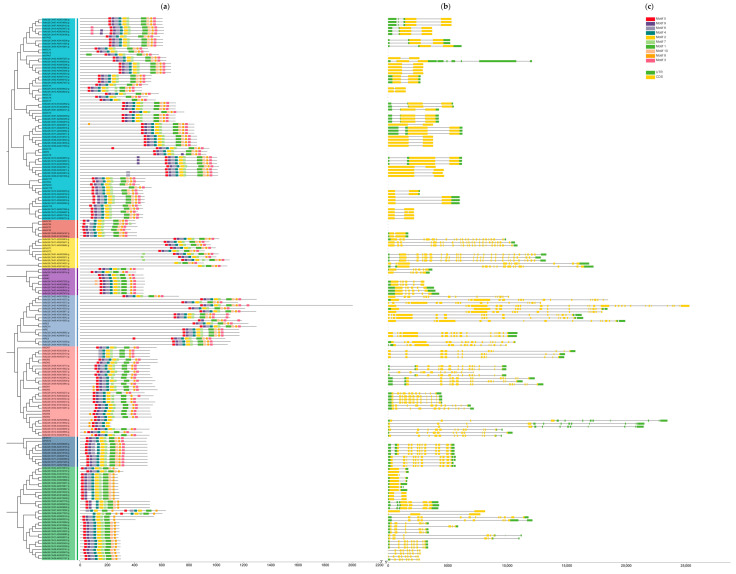
Phylogenetic relationships, conserved motifs, and gene structure of AGC family members. (**a**) Phylogenetic relationships of AGCs in potato and Arabidopsis thaliana. (**b**) The conserved motif distribution of AGCs. (**c**) Structure of exons and introns of AGCs. Exons and introns are indicated by rectangles and lines, respectively.

In order to better understand the *StAGC* gene family members, gene structure and conserved motifs analysis were performed in this study (Figure 2). All members share more than half of the conserved motifs, and the distribution of conserved motifs in the same subfamily is similar. AGC-1, AGC-2, AGC-2 relate, IRE, NDR, and PDK-1 subfamilies possess all motifs. As expected, genes in the same family share similar genetic structures. Members of subfamily S6K have similar sequence lengths, as do members of PDK-1. AGC2, AGC2 relate, S6K, IRE, NDR, PDK-1, and new subfamilies have more introns/exons. All genes of AGC1, which have the most members, have two CDS regions (Figure 2c).

### 3.3. Chromosome Distribution and Collinearity Analysis of AGC Family Genes in ‘Qingshu No. 9’

**Figure 3:**
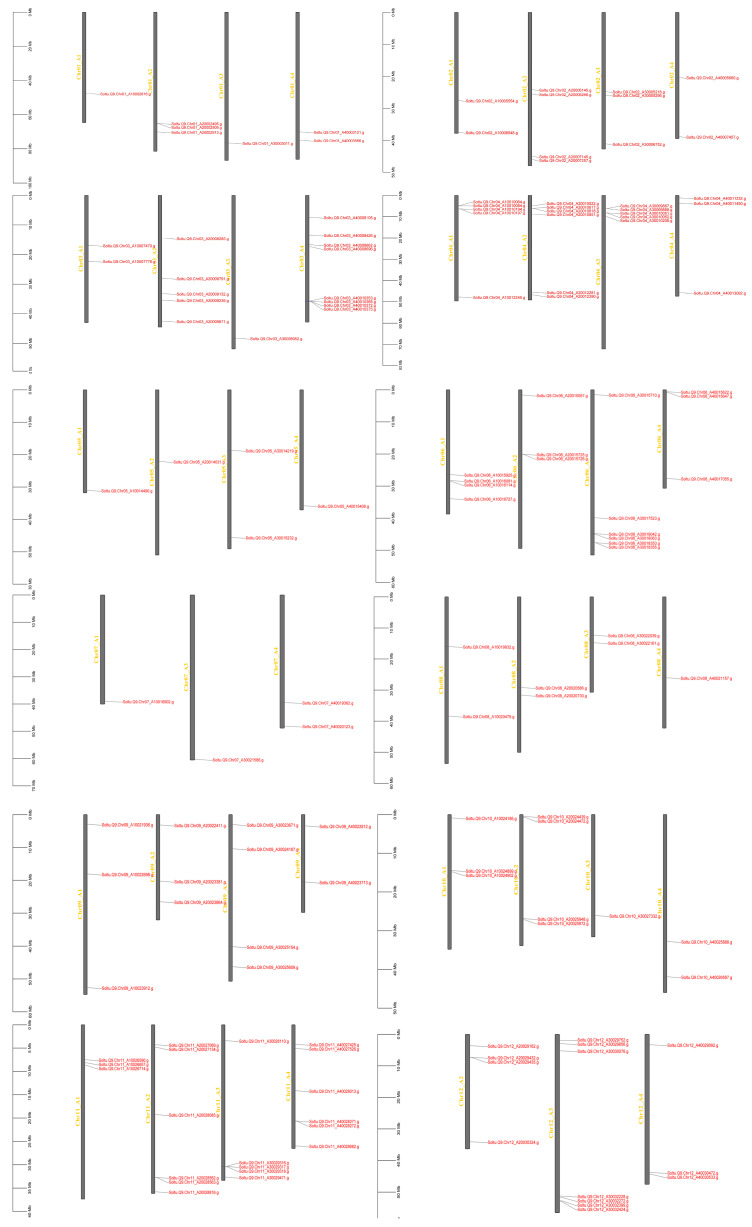
Distribution of AGC kinase family members on chromosomes of ‘Qingshu No. 9’.

**Figure 4:**
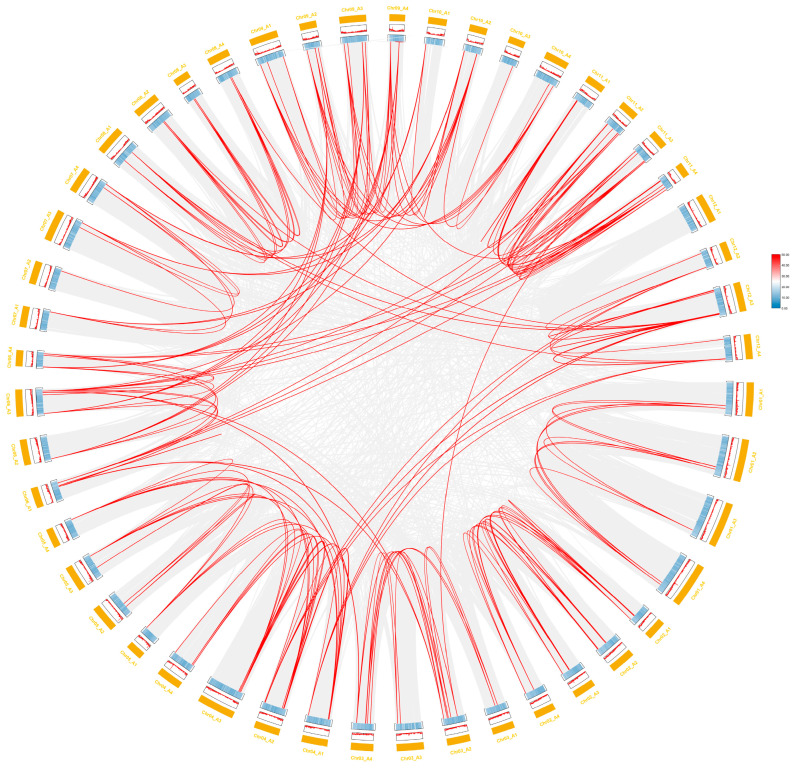
Collinearity analysis of AGC in the genome of ‘Qingshu No. 9’. In the potato variety ‘Qingshu No. 9’, 141 *StAGC* genes were located on 46 chromosomes. The grey lines in the background represent the collinear regions between the varieties of ‘Qingshu No. 9’, while the red lines indicate the collinearity of the AGC kinase gene pairs.

AGC gene family members are distributed on 46 chromosome arms. AGCs are not identified on chromosome 7 A2 and chromosome 12 A1, among which chromosome 11 has the most members, with 20 members; chromosome 7 has the fewest members, with 4 members (Figure 3). A total of 95 members of the *StAGC* gene family had gene duplication events, and 223 collinear pairs were identified, including 146 allelic pairs and 77 nonallelic pairs (Table S3). Six tandem duplications occurred in the identified gene duplication events (Table S3). In addition, the ratios of Ka and Ks values for 223 collinear pairs were calculated (Table S2), and Ka/Ks values for these 223 collinear pairs all showed <1 (Figure 4).

**Figure 5:**
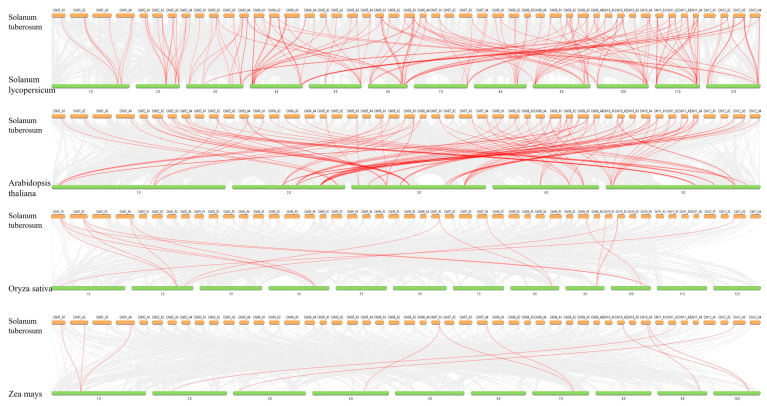
Collinearity analysis of AGC between the potato and different species. The gray lines in the background represent the collinear blocks between the potato and the other four plants, and the red lines show the collinearity of the AGC gene pairs.

The number of homologous gene pairs between the *StAGC* gene and tomato, *Arabidopsis thaliana*, rice, and maize was 192, 103, 17, and 11 (Figure 5), respectively, indicating that there was a close evolutionary relationship between potato and tomato, while the number of homologous genes among monocotyledonous plants decreased significantly. The number of homologous genes between potato and dicotyledonous plants was significantly higher than that between monocotyledonous plants.

### 3.4. Analysis of Cis-Acting Elements of AGC Gene Family in ‘Qingshu No. 9’

**Figure 6:**
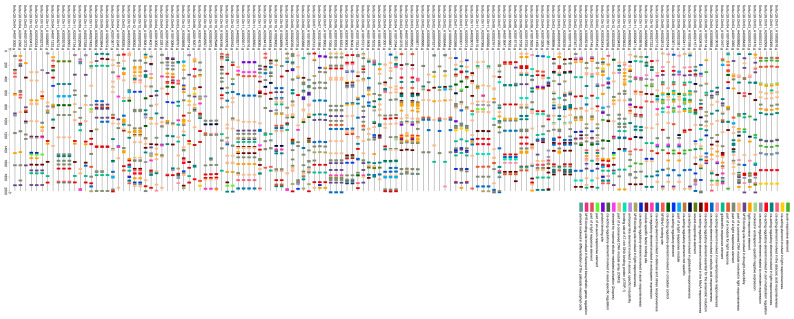
Analysis of cis-acting elements of potato AGCs. The differently colored boxes represent cis-acting elements, as indicated in the legend (lower right).

*StAGC* genes contain abscisic acid-inducing element, jasmonic acid-inducing element, salicylic acid-inducing element, auxin-inducing element, and gibberellin-inducing element (Figure 6). In addition, there are stress-related elements, such as hypoxia-specific induction response elements, drought response elements, low-temperature response elements, wound response elements, defense and stress response elements, and abundant light response elements. Of course, there are also growth and development-related elements, including meristem expression response elements, endosperm expression response elements, and palisade mesophyll cell response elements. Circadian rhythm regulatory elements and zein metabolic regulatory elements were classified into other groups. Among them, light response-related elements were the most numerous, followed by ABA-inducible elements and hypoxia-inducible elements.

### 3.5. Expression Patterns of AGC Gene Family Members in Different Tissues of ‘Qingshu No. 9’

**Figure 7:**
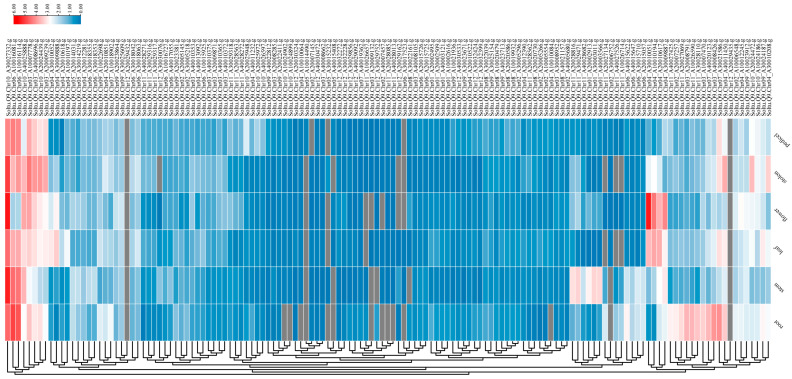
Tissue expression of *StAGCs* in ‘Qingshu No. 9’. The expression patterns within the organization involve six components: root, stem, leaf, flower, pedicel, and creeping stem. In the heat map, the darker the color, the higher the expression level of that component; the lighter the color, the lower the expression level.

Gene expression patterns are usually correlated with gene function. This study extracted FPKM values for six tissues from the biological project PRJCA006096 published by Wang, F. et al. [42] and subsequently mapped gene expression profiles (Figure 7). The results showed that *StAGC* gene family expression was significantly regulated in different tissues, and most of its members were expressed at low levels in the root, stem, leaf, flower, stolon, and pedicel. *Soltu.Q9.Chr10_A30027332.g*, *Soltu.Q9.Chr06_A10016081.g*, *Soltu.Q9.Chr06_A10016114.g*, *Soltu.Q9.Chr10_A40025888.g*, *Soltu.Q9.Chr03_A10007778.g*, *Soltu.Q9.Chr03_A40008696.g*, *Soltu.Q9.Chr10_A20024439.g*, and *Soltu.Q9.Chr03_A20009239.g* were highly expressed in six tissues, *Soltu.Q9.Chr04_A30010051.g*, *Soltu.Q9.Chr04_A10010194.g*, *Soltu.Q9.Chr04_A20010617.g*, and *Soltu.Q9.Chr04_A30009887.g* were highly expressed in flowers. *Soltu.Q9.Chr11_A20028816.g*, *Soltu.Q9.Chr11_A30029471.g*, *Soltu.Q9.Chr11_A40028682.g*, *Soltu.Q9.Chr01_A20002913.g*, *Soltu.Q9.Chr01_A30003011.g*, and *Soltu.Q9.Chr01_A40003566.g* were highly expressed in the stem, while low expression was observed in other tissues. *Soltu.Q9.Chr11_A40027425.g*, *Soltu.Q9.Chr02_A20007257.g*, *Soltu.Q9.Chr11_A20027069.g*, *Soltu.Q9.Chr03_A20008791.g*, *Soltu.Q9.Chr11_A10026590.g*, *Soltu.Q9.Chr11_A30028110.g*, *Soltu.Q9.Chr03_A10007470.g*, *Soltu.Q9.Chr07_A40020123.g* and *Soltu.Q9.Chr03_A40008426.g* were highly expressed in roots.

### 3.6. Expression Analysis of StAGCs Genes Under Potato Late-Blight Stress

**Figure 8:**
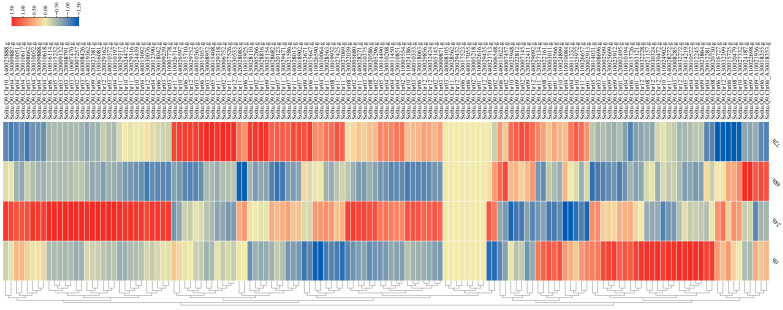
Expression patterns of *StAGCs* at different time points under late blight stress. This heat map shows the expression levels for four different time points. 0 h: the expression level before inoculation with late blight, 24 h: the expression level 24 h after inoculation with late blight, 48 h: the expression level 48 h after inoculation with late blight, and 72 h: the expression level 72 h after inoculation with late blight. The color is the key indicator: the redder the color, the higher the expression level; the bluer the color, the lower the expression level.

Approximately 78.1% of the members of the AGC kinase gene family exhibited a response to late-blight stress (Figure 8). At 24 h post-inoculation (hpi), compared to 0 hpi, 58 differentially expressed genes (DEGs) were identified, which included 39 upregulated and 19 downregulated genes. At 48 hpi, relative to 0 hpi, 35 DEGs were detected, comprising 9 upregulated and 26 downregulated genes. At 72 hpi, 61 DEGs were observed, consisting of 37 upregulated and 24 downregulated genes. In total, 10 genes were differentially expressed at all three time points (24 hpi, 48 hpi, 72 hpi) when compared to 0 hpi.

**Figure 9:**
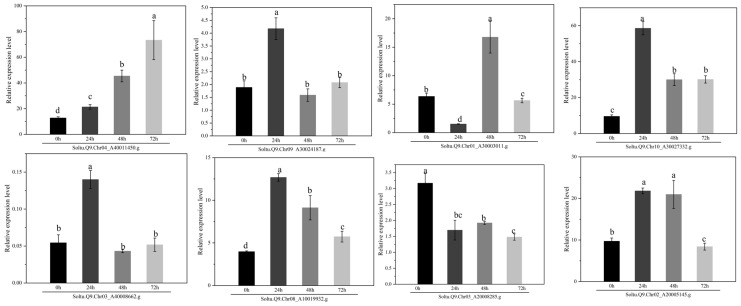
Expression of AGCs under late-blight stress at different times. The qRT-PCR was performed four times. Data are the mean ± SD of four biological repeats. Statistical significance was analyzed with a one-sided Kruskal–Wallis test with Bonferroni correction, followed by post hoc Dunn’s test. Data points with different letters indicate significant differences in *p* < 0.05.

In this study, one differentially expressed gene was selected from each of the eight subgroups within the AGC kinase family for validation through qRT-PCR (Figure 9). The quantitative fluorescence results confirmed that all eight selected genes responded to late-blight stress. The expression of the *Soltu.Q9.Chr10_A30027332.g* peaked at 24 h post-inoculation (hpi), exhibiting a 6.1-fold increase compared to 0 hpi. This finding is consistent with research by Wei, Y. et al. [11], which demonstrated that the *StS6K2* enhances potato resistance to late blight by regulating the WRKY59 transcription factor. The expression of the *Soltu.Q9.Chr04_A40011450.g* reached its maximum at 72 hpi, showing a 5.7-fold increase relative to 0 hpi. Wang et al. [57] identified *Soltu.Q9.Chr04_A40011450.g* as a candidate gene in their genome-wide association study (GWAS) of late-blight resistance traits among a natural population of 284 potato accessions. Furthermore, Nisha, B. et al. [58] reported significantly differential expression of the *Soltu.Q9.Chr04_A40011450.g* gene in resistant wild potato varieties after inoculation with the late blight pathogen, compared to uninoculated controls. Consequently, the *Soltu.Q9.Chr04_A40011450.g* (*StD6PK*) was selected for further analysis.

### 3.7. Subcellular Localization of StD6PK

**Figure 10:**
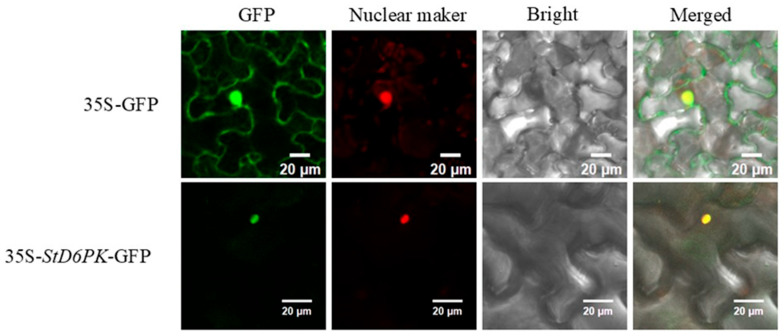
Subcellular localization results of the *StD6PK* gene. The *StD6PK*-*GFP* fusion vector enables the transient expression of the *GFP* gene driven by the CaMV-35s promoter in tobacco. The GFP signal (green) was co-localized with the nuclear localization signal (red), as shown in the fused fluorescence image (yellow).

The *StD6PK*-GFP vector was transformed into *Nicotiana benthamiana* using the transient expression method by fusion with GFP protein to determine the location of *StD6PK* in cell substructures. Fluorescence was observed by confocal microscopy. The *StD6PK*-GFP fluorescence signal was localized in the nucleus of Nicotiana benthamiana cells (Figure 10).

### 3.8. Overexpression of StD6PK Enhances Resistance to Late Blight in Potato

**Figure 11:**
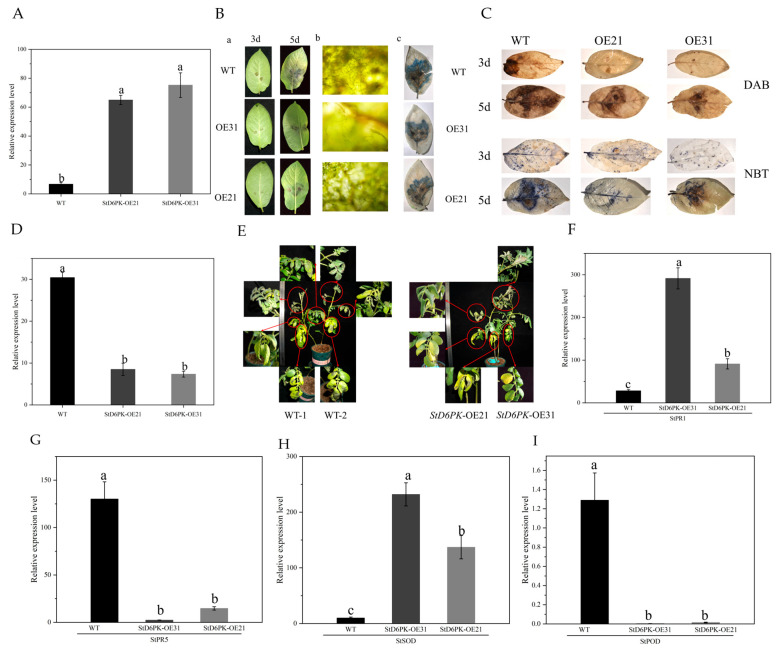
Expression quantification of transgenic *StD6PK*-OE lines and disease resistance assessment under late-blight stress. (**A**) The gene expression level of the *StD6PK* gene in transgenic lines and wild-type lines. (**B**) The disease development and trypan blue staining in wild-type and transgenic lines following *Phytophthora infestans* inoculation. (**Ba**) Disease symptoms of wild-type and OE lines at 3 days post-inoculation (dpi) and 5 dpi with *Phytophthora infestans*; (**Bb**) Microscopic observation of hyphal growth in wild-type and OE lines at 5 dpi; (**Bc**) Trypan blue staining of wild-type and OE lines at 5 dpi post-inoculation with late blight(*Phytophthora infestans*) pathogen. (**C**) These stainings represent DAB and NBT staining of wild-type and OE lines at 3 dpi and 5 dpi with *Phytophthora infestans*. (**D**) The percentage of lesion area in transgenic lines and wild-type lines. (**E**) This figure illustrates the phenotypic differences between wild-type and *StD6PK*-OE lines at 5 days post-inoculation with late blight. (**F**) Expression levels of *StPR1* in transgenic and wild-type lines. (**G**) Expression levels of *StPR5* in transgenic and wild-type lines. (**H**) Expression levels of *StSOD* in transgenic and wild-type lines. (**I**) Expression levels of *StPOD* in transgenic and wild-type lines. (**A**,**D**,**F**–**I**) Data are the mean ± SD of four biological repeats. Statistical significance was analyzed with a one-sided Kruskal–Wallis test with Bonferroni correction, followed by post hoc Dunn’s test. Data points with different letters indicate significant differences in *p* < 0.05.

To further evaluate the role of the *StD6PK* gene in enhancing resistance to late blight, we chose the top two overexpressing transgenic lines, OE31 and OE21, for additional analysis (Figure 11).

Three days after late blight inoculation, there was a significant difference in the lesion area between WT, OE31, and OE21 (Figure 11B). Five days after late blight inoculation (Figure 11D), the lesion area of WT was 4.14 times and 3.48 times that of OE31 and OE21, respectively. Moreover, under microscopic observation, the mycelium content of WT was significantly higher than that of OE lines and that of WT plants (Figure 11(Bb)). Five days post-inoculation with *Phytophthora infestans*, both overexpression (OE) lines exhibited milder disease symptoms compared to the wild-type (WT) plants, and late blight infection was observed on the upper, middle, and lower leaves of WT plants, while in OE lines, late blight infection was observed on the middle and lower leaves (Figure 11E). Moreover, in the proportion of leaves with lesion coverage exceeding 60%, WT lines exhibited a higher percentage relative to the total leaves compared to OE lines. This trend was also observed in leaves with lesion coverage ranging from 30%~60% (Table S6). Under unhandled conditions, significantly lower expression levels of *StPR1* and *StSOD* were observed in WT plants relative to OE lines, whereas *StPR5* and *StPOD* displayed significantly higher expression in WT plants (Figure 11F–I). This indicates that the overexpression of the *StD6PK* gene increases the resistance of potatoes to late blight. In addition, DAB, NBT, and trypan blue staining indicated that overexpression of *StD6PK* helps to reduce the accumulation of H_2_O_2_ and promote the integrity of the cell membrane (Figure 11C).

## 4. Discussion

The AGC kinase family, as an important subfamily of the protein kinase superfamily, plays a key role in plant growth and development. Its function was first revealed in *Arabidopsis thaliana*, involving lipid signaling pathways, cell proliferation, and auxin transport. However, research on this family in Solanaceae plants, particularly in potato, remains limited. Currently, the research on potato gene families mainly focuses on diploids, such as MYB, bHLH, bZIP, and other families [59,60,61]. However, common cultivars are often tetraploids with high genomic heterozygosity, and genetic analysis is relatively complex, so AGC gene families have not been studied in potato tetraploids. Until 2022, successive reports of the tetraploid potato C88.v1 genome and the tetraploid potato ‘Qingshu No. 9’ genome [42,62] provided the possibility for bioinformatics analysis and identification of the AGC gene family on the whole tetraploid potato genome. This study identified 141 AGC members in potato for the first time, which is more than the number of members reported in the diploid plants *Arabidopsis thaliana* and rice [3,7].

Phylogenetic analysis of AGC proteins across various species revealed that *StAGC* family members could be categorized into eight subfamilies, one additional compared to dicotyledonous *Arabidopsis*, and two more than monocotyledonous rice. The NDR subfamily was absent in rice [3]. These findings suggest that the AGC family likely evolved after the divergence of monocotyledons and dicotyledons, with tetraploids potentially generating new members through natural duplication events.

Analysis of conserved motifs revealed distinct types among different subfamilies, contributing to functional diversity over evolution. Within the same subfamily, shared conserved motifs indicate functional similarities. Gene structure diversity likely influences the evolution of gene families [63]. Examination of introns demonstrated significant variation in the number of exons and introns across subpopulations. AGC1 and AGC2 subpopulations exhibited the lowest count of 1~2 introns. In contrast, IRE and NDR subpopulations displayed numerous exons and introns. For instance, *SoltuQ9.Chr03_A30008952.g* in the IRE subpopulation contained 28 introns. Discrepancies in exon counts suggest substantial divergence within the *StAGC* gene family throughout prolonged evolution.

Cis-acting elements serve as binding sites for transcription factors that regulate gene expression processes [64,65]. The promoter region of the *StAGC* gene analyzed in this study exhibited a high abundance of light-responsive elements, suggesting its potential involvement in the photoregulation of tetraploid potato. Furthermore, *StAGC* harbors numerous elements associated with hormones, anaerobic conditions, low temperatures, and drought stress. Some elements within *StAGC* also include MYB binding sites linked to the regulation of flavonoid biosynthesis, indicating its dual role in responding to biotic and abiotic stresses, as well as potentially participating in the control of flavonoid synthesis.

It is well known that gene duplication events (segmental duplication, WGD, and tandem duplication) play an important role in biological evolution [66]. In this study, most gene pairs in the AGC gene family exhibited Ka/Ks ratios less than one, indicating that purifying selection played a major role in the evolution of these genes, suggesting that mutations within these genes are generally detrimental to protein function. A total of 95 out of 141 *StAGC* genes had gene duplication events during evolution, and most of the gene duplication events were segment/whole genome duplication; only 6 pairs were tandem duplication. Therefore, whole genome duplication (WGD) and segment duplication were the main driving forces. This is different from the AGC family replication events identified by Jiang et al. [3] in rice, where all replication events are segment/whole genome replication. These gene duplication events promoted the amplification and functional diversity of AGC gene family members in tetraploid potato. In this study, the number of AGC gene collinear pairs between potato and tomato was up to 192, indicating that there was a close evolutionary relationship between potato and tomato, while the number of homologous genes between monocotyledonous plants showed a significant decline. Recently, Huang Sanwen’s team confirmed that potato is a hybrid of tomato as the female parent and a potato-like plant as the male parent [67]. These results suggest that there may be more gene duplication events in dicotyledonous plants during evolution.

Gene expression patterns are closely linked to biological function. Eight *StAGC* genes (e.g., *Soltu.Q9.Chr10_A30027332.g*) showed high expression across six tissues. Zhang et al. [68] reported that the homologous genes *ATPK1* and *ATPK2* are associated with *Q9.Chr10_A30027332.g*, *Soltu.Q9.Chr06_A10016081.g*, *Soltu.Q9.Chr06_A10016114.g*, and *Soltu.Q9.Chr10_A40025888.g* are involved in the regulation of plant growth and development. Their expression was detected across all tissues and developmental stages. It is hypothesized that genes exhibiting high expression in diverse tissue types may be essential for the regulation of plant growth and development. Four flower-specific genes (e.g., *Soltu.Q9.Chr04_A30010051.g*) are part of the PEPCK (Phosphoenolpyruvate carboxykinase), which supplies lipids, proteins, and carbohydrates [69], potentially impacting floral organ development and pollination. Six genes highly expressed in stems, namely *Soltu.Q9.Chr11_A20028816.g*, are associated with the photoreceptor PHOT1/PHOT2, known to play a role in the phototropic growth of plants [70]. Nine genes, including *Soltu.Q9.Chr11_A40027425.g*, exhibit high expression levels in roots. It has been observed that the homologous genes *AtPDK1* of *Soltu.Q9.Chr11_A40027425.g*, *Soltu.Q9.Chr11_A20027069.g*, *Soltu.Q9.Chr11_A10026590.g*, and *Soltu.Q9.Chr11_A30028110.g* regulate the growth of Arabidopsis root hairs [71]. Additionally, *Soltu.Q9.Chr11_A40027425.g* and *Soltu.Q9.Chr07_A40020123.g* are involved in modulating the polar transport of auxin [72,73], a process crucial for root development [74,75]. In summary, the distinct expression patterns of the *StAGC* gene across various tissues imply that its function may have evolved in response to diverse environmental factors.

The expression patterns of *StAGCs* during Phytophthora infections provide valuable insights into their potential roles in potato defense mechanisms. Transcriptome analysis revealed that 78.1% of the family members responded to late-blight stress, indicating a significant involvement of the AGC kinase gene family in late-blight response, consistent with findings reported by He, M. et al. [49]. qRT-PCR validation confirmed that all eight selected genes responded to late-blight stress, with Gene 1 and Gene 2 exhibiting 6.1-fold and 5.7-fold upregulation, respectively. Further studies demonstrated that *Soltu.Q9.Chr10_A30027332.g* enhances potato resistance to late blight by modulating the transcription factor *WRKY59* [11]. Wang et al. [57] identified *Soltu.Q9.Chr04_A40011450.g* as a candidate gene in their genome-wide association study (GWAS) of late blight resistance traits among a natural population of 284 potato accessions. Nisha, B. et al. [58] reported significantly differential expression of the *Soltu.Q9.Chr04_A40011450.g* gene in resistant wild potato varieties after inoculation with the late blight pathogen, compared to uninoculated controls. Furthermore, Zhang et al. [76] demonstrated that overexpression of *D6PKL2* enhances resistance to Fusarium wilt in Paulownia trees. These findings collectively indicate that the AGC kinase gene family plays an essential role in plant defense against biotic stresses.

Potato plants afflicted by late blight often suffer near-total yield loss, underscoring the urgency of identifying disease-resistant genes and developing resistant varieties in potato breeding. This study employed quantitative real-time PCR (qRT-PCR) to analyze late blight-inoculated potato plants for responsive AGC genes. Among these genes, *StD6PK*, showing notable upregulation post-pathogen exposure, was singled out for further scrutiny. Subsequent overexpression analysis of transgenic potato lines (*StD6PK*-OE) demonstrated that, in comparison to wild-type plants, OE plants exhibited a reduced lesion area, alleviated overall disease symptoms, decreased hyphal biomass, lower H_2_O_2_ levels, and fewer instances of cell death. Additionally, the expression levels of *StPR1* and *StSOD* were upregulated, while those of *StPR5* and *StPOD* were downregulated. These findings indicate that overexpression of *StD6PK* enhances potato resistance to late blight, potentially in association with *StPR1*, *StPR5*, *StSOD*, and *StPOD*. *StPR1* serves as a canonical salicylic acid (SA)-dependent marker gene for systemic acquired resistance, and its upregulation indicates the activation of the SA pathway [77]. Conversely, *StPR5* is typically induced under conditions of severe infection [78]. Therefore, it is hypothesized that the overexpression of *StD6PK* in this study triggered the activation of the SA pathway, leading to a significant induction of *StPR1*. Given that the samples analyzed were untreated wild-type and OE (overexpression) plants, *StPR5* was not strongly induced. The observed increase in *StSOD* expression may result from the overexpression of *StD6PK*, which activates the salicylic acid (SA) pathway [79]. SOD catalyzes the conversion of superoxide anions into H_2_O_2_, a crucial signaling molecule in the SA pathway during the initial stages of disease resistance. However, POD is responsible for the removal of H_2_O_2_. To mitigate the rapid clearance of H_2_O_2_, the SA signaling pathway may downregulate the transcription of specific POD genes, consequently leading to a reduction in the expression levels of *StPOD* genes [80]. Previous studies have indicated that *D6PK* is involved in auxin transport [81,82,83]. It is thus posited that *StD6PK* may modulate salicylic acid (SA) pathways by participating in the indole-3-acetic acid (IAA) pathway, thereby contributing to potato interactions with *P. infestans* [84]. In future studies, we will conduct loss-of-function assays for *StD6PK* to further validate its role, followed by screening for interacting genes to delineate the specific genes regulated by *StD6PK* during its interaction with *Phytophthora infestans*.

## 5. Conclusions

In this study, the AGC kinase family in the tetraploid potato ‘Qingshu No. 9’ genome was analyzed comprehensively for the first time. A total of 141 *StAGC* genes were identified, distributed unevenly on 46 chromosome arms and divided into eight subfamilies. Collinearity analysis showed that whole-genome duplication and segmental duplication were the main driving forces for family expansion, and purification selection was the main driving force for family evolution. Abundant light-responsive elements and hormone-inducible elements were detected, and some genes responding to late blight were found, indicating that this family may be related to resistance mechanisms to late blight. qRT-PCR was performed on eight selected *StAGCs*, and eight members were identified to show a strong response to late-blight stress, indicating that they have a role in late-blight stress. Subcellular localization analysis revealed that *StD6PK* is localized to the nucleus. Overexpression of this gene reduced H_2_O_2_ levels in leaves, decreased cell death, and enhanced potato resistance to *Phytophthora infestans*. It is further hypothesized that *StD6PK* may participate in potato *P. infestans* interactions by modulating the salicylic acid (SA) pathway through its involvement in the indole-3-acetic acid (IAA) pathway. Taken together, this study broadens our understanding of the AGC kinase family, provides valuable insights for elucidating the functions of AGC genes in potato, and lays a foundation for breeding late blight-resistant potato cultivars.

## Data Availability

All data generated or analyzed during this study are included in this published article and its additional files. The RNA-Seq data are available in the BioProject accession: PRJCA006877 repository, http://bigd.big.ac.cn, accessed on 25 June 2025.

## Supplementary Materials

The following supporting information can be downloaded at: https://www.mdpi.com/article/10.3390/plants14243818/s1, Table S1: Basic information of StAGC family genes and proteins in ‘Qingshu No. 9’; Table S2: Ka/Ks ratios of StAGC genes in tetraploid potato; Table S3: Gene duplication of StAGC genes; Table S4: Primer sequences used in this study; Table S5: Expression Patterns of StAGCs at Different Time Points Under Late Blight Stress; Table S6: Phenotypic statistics of WT and OE strains 5 days after inoculation with late blight. Figure S1: The vector used for overexpression and the enzyme digestion of *StD6PK*; Figure S2: The growth pictures of *DH5α* transformed by PRI101-*StD6PK* recombinant vector and PCR pictures of monoclonal bacteria solution; Figure S3: The process of transforming potato ‘Qingshu No. 9’ with the recombinant vector; Figure S4: The PCR-based confirmation of transgenic regenerated plants expressing *StD6PK*.
