# Supplementary material for: Genome-Wide Identification of the AGC Kinase Family in Tetraploid Potato (Solanum tuberosum L.) Cultivar ‘Qingshu No. 9’ and Functional Analysis of StD6PK in Response to Late Blight (Phytophthora infestans)
Source: Plants (Basel). 2025 Dec 15;14(24):3818. doi: 10.3390/plants14243818 (PMC12737297; doi:10.3390/plants14243818)
Supplement: Supplementary file 1 [file plants-14-03818-s001.zip › Fig. S.pdf]

a

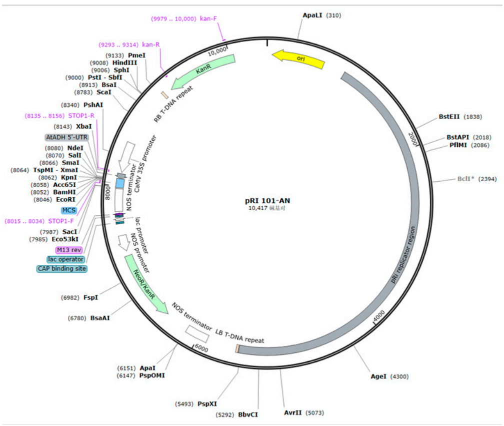

b

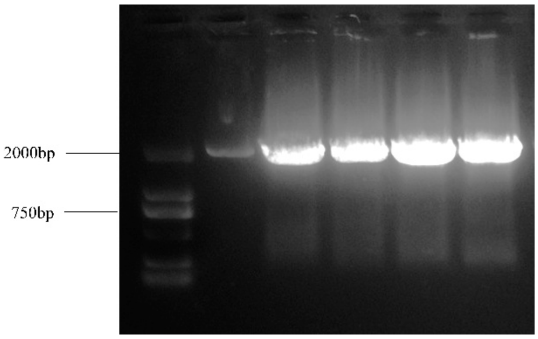

Figure S1. The vector used for overexpression and the enzyme digestion of *StD6PK*.

a. Map of overexpression vector PRI101, b. Gel electropherogram of *StD6PK* post-digestion purification using DL2000 DNA marker (indicating positions), with *StD6PK* fragment length of 1824 bp.

a

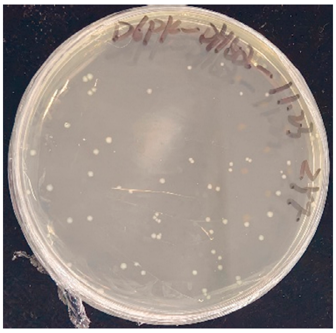

b

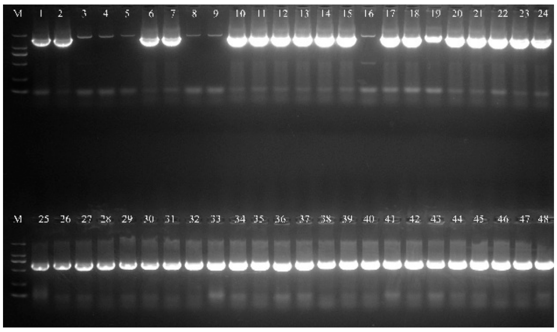

Figure S2. The growth pictures of *DH5α* transformed by PRI101-*StD6PK* recombinant vector and PCR pictures of monoclonal bacteria solution.

a. Growth image of E. coli *DH5α* transformed with PRI101-*StD6PK* recombinant vector. b. PCR results of monoclonal bacterial liquid picked from a using *StD6PK*-specific primers and kanR-specific primers, Lane M is DL2000 DNA Marker. The size of the kanR fragment is 705 bp.

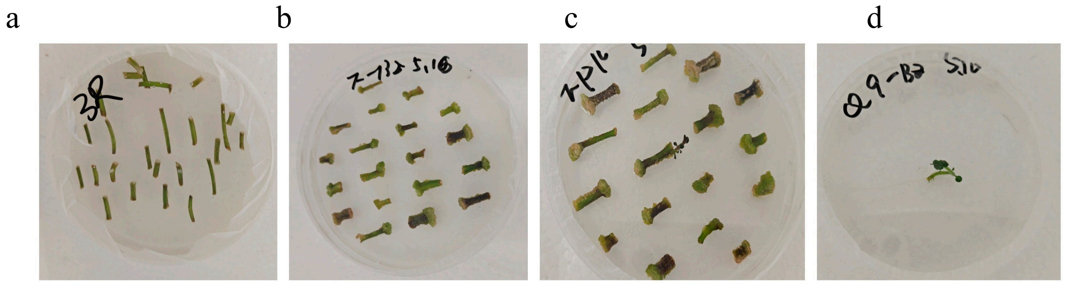

Figure S3. The process of transforming potato 'Qingshu No. 9' with the recombinant vector. a. Image of 'Qingshu No. 9' stem segments placed on co-cultivation medium after immersion in Agrobacterium tumefaciens containing the recombinant vector. b. Photograph showing stem segments transferred to callus induction medium with initial swelling. c. Regenerated shoots emerging from callus differentiated from stem segments. d. Image of regenerated shoots cultured on rooting medium until root formation.

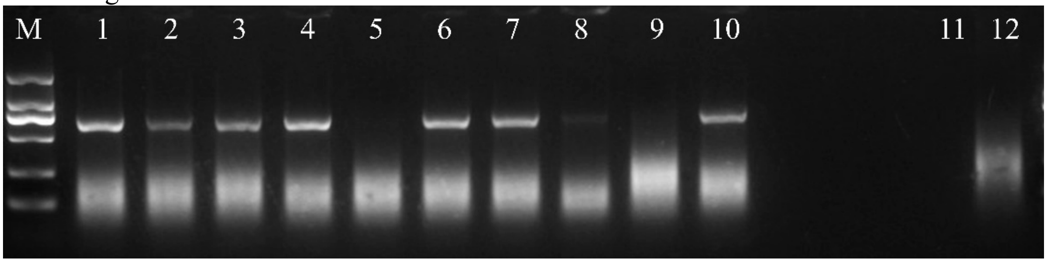

Figure S4. The PCR-based confirmation of transgenic regenerated plants expressing *StD6PK*. Lane M: DL2000 DNA Marker, Lanes 1~10: Independent transgenic regenerated lines, Lane 11: Negative control (water as template), Lane 12: PCR amplification using wild-type genomic DNA as template.
